# Draft genome sequence of the basidiomycetous yeast *Kockovaella fuzhouensis* CBS 8243

**DOI:** 10.1128/mra.00426-26

**Published:** 2026-06-24

**Authors:** Hiroki Okada, Lin Feng, Xi Chen, Erfei Bi

**Affiliations:** 1 Department of Cell and Developmental Biology, Perelman School of Medicine, University of Pennsylvania 6572, Philadelphia, Pennsylvania, USA; 2 Fisheries College, Sichuan Agricultural University 506176, Chengdu, Sichuan, China; University of California Riverside, Riverside, California, USA

**Keywords:** basidiomycetous yeast, *Kockovaella fuzhouensis* CBS 8243, long neck yeast, genome sequence

## Abstract

We report the draft genome sequence of the basidiomycetous yeast *Kockovaella fuzhouensis* CBS 8243. The 23.2 Mb genome (54.2% GC) comprises 8 scaffolds (N50, 3.2 Mb) and 8,593 predicted genes, with 95.2% BUSCO completeness. This genome provides a resource for studies of cell morphogenesis and division.

## ANNOUNCEMENT

*Kockovaella fuzhouensis* (formerly *Fellomyces fuzhouensis*) is a basidiomycetous yeast originally isolated from mango flowers in Fuzhou, China (26.0614 N 119.3061 E) (1–6). Although its genome sequence has not been previously reported, this yeast has been studied for its distinctive morphology and mode of cell division. Notably, it forms a long, narrow neck through which the nucleus migrates from the mother to the daughter (or conidium) prior to mitosis, followed by cytokinesis at the base of the daughter cell (7, 8). Here, we report the draft genome sequence to facilitate genetic and cell biological analyses of these fundamental processes.

Strain CBS 8243 was obtained from the CBS culture collection (Westerdijk Fungal Biodiversity Institute, Netherlands) and maintained under standard laboratory conditions. Cells were cultured overnight in YM-1 liquid medium supplemented with 2% glucose in baffled Erlenmeyer flasks at 25°C with shaking at approximately 200 rpm. Cells were harvested by centrifugation (8,000 × *g*, 4°C, 20 min), and the pellet was resuspended in SLS extraction buffer (0.58% NaCl, 1% sodium lauroyl sarcosinate, 0.1 M Tris-HCl, 20 mM EDTA, pH 8.0). Cell lysis was performed by five freeze-thaw cycles, followed by incubation at 23°C for 2 h. Genomic DNA was then purified by standard phenol-chloroform-isoamyl alcohol extraction.

Whole-genome sequencing was performed by Novogene (Beijing, China) using the Illumina HiSeq 2500 platform, generating 150-bp paired-end reads from libraries with insert sizes of approximately 350 bp, 1.5 kb, and 6.5 kb. Approximately 2.9, 2.2, and 1.9 Gb of raw data were obtained from the respective libraries (7.0 Gb in total). Raw reads were quality filtered and adapter trimmed using the standard quality-control pipeline provided by Novogene prior to *de novo* assembly. Approximately 8.9, 3.9, and 3.0 million read pairs were retained, corresponding to 2.7, 1.1, and 0.9 Gb of clean data, respectively. In total, 4.7 Gb of clean data were obtained, providing ~200× genome coverage.

*De novo* assembly was performed using SOAPdenovo (9, 10) with the standard assembly pipeline provided by Novogene. The initial assembly consisted of 293 contigs organized into 12 scaffolds. During the NCBI submission, four short scaffolds were identified as likely contaminants and removed. The final draft genome assembly comprised 288 contigs arranged in 8 scaffolds, with a total assembly size of 23.2 Mb, a scaffold N50 of 3.2 Mb, and a GC content of 54.2%.

Genome completeness was assessed using BUSCO v6.0.0 with the *basidiomycota_odb10* data set (1,764 markers; 11, 12) in combination with MetaEuk-based gene prediction, yielding a completeness score of 95.2%. A total of 8,593 protein-coding genes were predicted using GeneMark-ES v4.72 in fungal mode (13, 14).

## Data Availability

This Whole Genome Shotgun project has been deposited at DDBJ/ENA/GenBank under accession number JBWVZG000000000. The version described here is JBWVZG010000000. Sequencing reads are available in the Sequence Read Archive (SRA) under accession number PRJNA1446923.
